# Quality of Life and Health Determinants of Informal Caregivers Aged 65 Years and Over

**DOI:** 10.3390/epidemiologia4040039

**Published:** 2023-11-06

**Authors:** Fanny Buckinx, Stéphane Adam, Mylène Aubertin-Leheudre, Marie De Saint Hubert, Alexandre Mouton, Florence Potier, Jean-Yves Reginster, Olivier Bruyere

**Affiliations:** 1 WHO Collaborating Center for Public Health Aspects of Musculo-Skeletal Health and Ageing, Division of Public Health, Epidemiology and Health Economics, University of Liège, 4000 Liège, Belgium; jyreginster@uliege.be (J.-Y.R.); olivier.bruyere@uliege.be (O.B.); 2 Psychology of Aging Unit, University of Liège, 4000 Liège, Belgium; stephane.adam@uliege.be; 3 Département des Sciences de L’activité Physique & Centre de Recherche de L’institut Universitaire de Gériatrie de Montréal, Faculté des Sciences, Université du Québec à Montréal, Montréal, QC H3W 1W5, Canada; aubertin.mylene@gmail.com; 4 Department of Geriatric Medicine, CHU UCL Namur, Institut de Recherche Santé Société, UCLouvain, 5530 Yvoir, Belgium; marie.desainthubert@chuuclnamur.uclouvain.be (M.D.S.H.); florence.potier@gmail.com (F.P.); 5 Research Unit for a Life-Course Perspective on Health & Education—RUCHE, Department of Sports Sciences, University of Liège, 4000 Liège, Belgium; alexandre.mouton@uliege.be

**Keywords:** informal caregivers, survey, health determinants, physical activity, older people

## Abstract

Informal caregivers’ own quality of life, health status, and determinants are poorly understood despite their concern for the health of the individuals they assist. To compare the quality of life and the health determinants of older informal caregivers with those of older adults without caregiving responsibilities. An online survey was designed to investigate the quality of life and the health determinants of people aged 65 years and over, with a focus on informal caregivers. In addition to socio-demographic data, the number of informal caregivers was ascertained and the Zarit scale of caregiver burden was applied. Quality of life (SF-12) and health determinants (access to technology and level of physical activity (IPAQ)) were assessed and compared between informal caregivers and non-caregivers. A total of 111 participants were included in the study (70 ± 3.83 years, 71.2% women). The majority of respondents (91.8%) were Belgian. One-third of the respondents identified themselves as informal caregivers and declared themselves as having a severe burden (61.9 ± 15.2/88). Socio-demographic characteristics and access to technology were similar between informal caregivers and non-caregivers (*p* > 0.05). However, informal caregivers had a lower SF-12 score in the mental score domain (44.3 ± 10.2 vs. 50.7 ± 7.0; *p* = 0.004) and a lower level of physical activity (434 ± 312 METS/min/week vs. 1126 ± 815 METS/min/week; *p* = 0.01) than their peers. Informal caregivers reported a lower quality of life and a lower level of physical activity than their peers. Given the recognized importance of physical activity for overall health, this survey highlights the need to promote physical activity among older informal caregivers.

## 1. Introduction

Informal care is defined as any assistance that is provided to a person in need of care by someone in the person’s direct environment [1]. It also includes less intensive assistance, assistance given to household members, and assistance given to institutionalized people. Informal care activities include emotional support, administrative help, advice on making appointments, transport assistance, and domestic and personal care. This informal care is unpaid, results from social rather than professional relationships, and involves long-term care for sick family members or friends [1]. Informal care plays an important role in the care of people with health problems. There is, therefore, a considerable public interest in this type of care in Europe [2].

In Europe, around a third of the population is considered to be informal caregivers (34.3%), but variations between countries are large (from 43.6% in Finland to 8.2% in Hungary) [3]. The caregivers are mostly aged between 50 and 75 years and help their parents or partners. Informal caregivers are more often women, especially daughters or daughters-in-law [4]. The care-receivers can be older people with a loss of autonomy but also include sick or disabled children or young adults. The age of the care-receiver is mostly 75 years old and over [3]. The factors that encourage people to become informal caregivers are emotional ties (e.g., love and affection), a sense of duty, and a personal sense of obligation [5].

Overall, caregiving is not associated with negative health effects [6]. A systematic review of the literature even found positive effects due to short-term informal care on self-reported physical health [7]. The positive impact of informal care on self-assessed health could be the result of a bias related to reference points, as argued by Di Novi and colleagues (2015). Spending time with someone with poor health might raise self-rated health assessments because people might compare their own health status to the poor health of the care recipient, although the objective health of the carer might be lower [8]. Unfortunately, being an informal caregiver over a long period of time is not without constraints, as this role is recognized as being a major cause of stress. In fact, these individuals must deal with the deteriorating health of the care-receiver, which, in turn, puts their own health at risk [9]. Indeed, informal caregivers are more likely to report symptoms of depression and other indicators of psychological distress than non-informal caregivers [10,11,12]. Furthermore, informal caregivers have poorer physical health than those who do not fulfill this role [10,13]. Several factors increase the caregiver burden, including high levels of disability and morbidity in the care-receiver, the number of hours of care, high variability in care tasks, the care setting (home care vs. institutional care), and the sex and age of the informal caregiver [14,15]. Nonetheless, while informal caregivers often feel burdened by caring for a person, they can also experience positive outcomes from caring; these are known as caregiver gains. One of these relatively overlooked caregiver benefits is increased self-esteem [16].

It is acknowledged that providing informal care has a negative impact on the caregivers’ quality of life. QoL, as a multidimensional concept, combines several aspects of health, such as physical, mental, emotional, and social functioning [17,18]. Comparing the QoL of informal carers with that of the general population is of great scientific importance. Such comparisons allow us to understand the unique challenges and burdens that caregivers face, shed light on potential health inequalities, and develop targeted interventions to improve their well-being. In addition, examining differences in quality of life can contribute to the development of policies aimed at providing adequate support and resources for this important group of people.

In addition, several determinants of healthy aging have been identified. An important one is physical activity. Indeed, the health benefits of physical activity are well recognized and are related to the following outcomes: mortality, cognitive status, physical autonomy, glycaemic control, pain, disability, muscle and bone strength, depression, and well-being [19,20,21,22]. According to the systematic review by Lindsay et al., previous research has shown that informal carers have low levels of physical activity and are at higher risk of being physically inactive than those who do not provide care, while other research has suggested that informal carers may have higher levels of physical activity than the general population, due to the physical demands of caring [23]. Overall, in order to develop appropriate interventions and policies to promote the health and well-being of carers, there is a need to better understand their physical activity levels.

The link between the social network and health has received additional attention in recent years [24]. In this sense, internet connectivity (i.e., access to technology) is a social determinant of health, as it can support a range of health information needs [25]. The use and access of tools such as patient portals, health trackers, health applications, and remote monitoring devices are increasing. This phenomenon is related to research findings suggesting that these tools can promote greater patient engagement, offer better support for patients outside of the usual care system, and improve health parameters [25]. In this sense, some authors have highlighted that computers and mobile devices are practical platforms for delivering care-related information and support services to informal carers, but that their reach may be more limited for carers who are older, less educated, and less healthy [26]. Understanding the digital divide among older informal caregivers and other carers can help identify barriers and develop targeted interventions to bridge the gap, ensuring that they have equal access to technological resources, information, and support systems, ultimately improving their caregiving experience and overall well-being.

Moreover, ageism (i.e., ageism is the umbrella term for stereotyping, prejudice, and discrimination against people because of their chronological age or the perception that they are “too old” or “too young” to be doing something [27]) is another determinant of worse health [28]. For example, ageism reduces life expectancy, worsens physical and mental health, hinders recovery from disability, and accelerates cognitive decline [28]. Ageism also increases social isolation and loneliness and reduces access to employment, education, and healthcare [28]. However, all these aspects are also known to affect health [28]. Based on the literature, the beginning of informal care is significantly associated with a better attitude toward old age, while the end of informal care is significantly associated with an increase in subjective age and an earlier onset of old age [29]. Measuring the self-perception of aging among informal caregivers is vital for understanding their attitudes and expectations regarding their caregiving role. This helps identify age-related biases, assess well-being, and develop interventions to promote positive aging experiences, enhancing the overall quality of life.

Health literacy is also one of the determinants of health since low health literacy is associated with more hospitalizations, greater use of emergency care, lower use of preventive services, poorer ability to interpret labels and health messages, poorer health outcomes, higher mortality, and higher healthcare costs [30,31,32]. To play their role properly, informal carers need to be able to access and understand information about their patient’s health, to establish valuable communication with both the patient and the healthcare providers, and to manage the services offered by the healthcare system [32]. By measuring health literacy, we can develop targeted interventions to improve caregivers’ health-related knowledge and empower them to actively engage in healthcare decision-making, ultimately enhancing their caregiving effectiveness and the well-being of care recipients.

Finally, a sense of coherence is a strong determinant of positive health [33,34]. In fact, the sense of coherence is not only associated with positive well-being, mental health, and quality of life [35] but also with the reduced severity of anxiety and depression [35]. It is an important determinant of the well-being of informal caregivers and may protect them from high levels of psychological distress and caregiver burden [36]. By measuring the sense of coherence, we can identify areas for support and intervention, enhance the caregiver’s psychological well-being, and develop strategies to strengthen their ability to manage stress and maintain a sense of purpose in their caregiving role. This measurement is essential for promoting caregivers’ overall resilience.

However, the quality of life and these health determinants have rarely been compared between older informal caregivers and the general older population. The aim of this study is, therefore, to fill this gap by assessing the quality of life and the health determinants of older informal caregivers and by comparing their profiles with those of their peers.

## 2. Methods

### 2.1. Study Design

The study was performed via an online survey carried out between August and October 2022, using the “Sondage Online” software, Available online: www.sondageonline.com (accessed on 15 July 2022). The protocol of this study has been approved by the hospital faculty ethics committee of the of the University of Liège (number 2022/339).

### 2.2. Study Population

All people aged 65 years and over were invited to participate in the survey. No exclusion criteria were defined. Participants were recruited via social networks (posting the link to the online survey on the co-authors’ social media) and via neurologic and geriatric consultation at the CHU XXX. In addition, during the special week dedicated to informal caregivers (10 to 14 October 2022) in Belgium, the authors attended various events dedicated to informal caregivers to invite them to participate in the survey. In fact, the authors participated in a discussion group (Heusy, Belgium), a “café aidant-proche” organized by the “Ligue Alzheimer” (Huy, Belgium), offering a day of support, help, and advice for informal caregivers, along with an interactive show (Fléron, Belgium) and an online information session organized by “Le Réseau Santé Bruxellois”. A convenience sample was, therefore, selected.

### 2.3. Data Collected

#### 2.3.1. Data Collection

##### Socio-Demographic Data

Data comprising the respondents’ age, sex, body mass index (BMI), country, marital status (i.e., married, a bachelor, widower, or divorced/separated), place of residence (i.e., house, apartment, or other residence), the number of people living with the respondent, the highest level of education (i.e., university, higher education, upper secondary education, lower secondary education, primary education, or none), monthly household income, smoker (i.e., yes or no), number of alcoholic drinks per day, number of chronic diseases, number of medicines taken per day, and number of vitamins or food supplements taken per day were collected to characterize the population sample.

##### Informal Caregivers

In order to identify the informal caregivers, the following question was asked: “Do you regularly help a person with a loss of autonomy (i.e., a person who is unable to perform alone certain activities of daily living)?”.

Those who answered “yes” to this question were asked to answer questions about the help provided: who was the care-receiver (i.e., the spouse, a parent/grandparent, a child, a friend, a neighbor, or other); whether they lived with the care-receiver, or the distance between the care-receiver’s home and the informal caregiver’s home; the age of the care-receiver; tasks performed to support the care-receiver (i.e., basic needs, meals, housekeeping, laundry, budget management, administrative management, medication management, work, hobbies, relationships, transportation, and supervision); what was the care-receiver suffering from (i.e., physical difficulties, mental difficulties, or both); time spent per week with the care-receiver; personal motivations to support the care-receiver (i.e., love/affection/friendship, recognition, the challenge, the obligation, keeping a promise, duty, religious beliefs, financial constraints, or other); whether the respondent was the only one caring for this person. All these questions were closed questions. In addition, the Zarit scale, which measured the caregivers’ burden, was also employed [37]. This questionnaire included 22 items and the total score ranged from 0 to 88 (0–21: no to mild burden; 21–40: mild to moderate burden; 41–60: moderate to severe burden; ≥61: severe burden) [37].

##### Quality of Life

The validated Short-Form-12 (SF-12) questionnaire was also used to assess the quality of life [38]. The SF-12 is a self-reported outcome that assesses the impact of health on an individual’s daily life. The SF-12 uses the following eight domains: (1) limitations in physical activities due to health problems; (2) limitations in social activities due to physical or emotional problems; (3) limitations in the usual role and activities because of physical health problems; (4) bodily pain; (5) general mental health (psychological distress and well-being); (6) limitations in the usual role and activities due to emotional problems; (7) vitality (energy and fatigue); and (8) general health perceptions. An algorithm calculated one score out of 100 for the physical score and another one for the mental score [38]. A higher score indicates a better quality of life.

##### Access to Technologies

Participants were asked about the frequency of their internet use, their ownership of connected devices (i.e., smartphone, tablet, laptop, computer), their use of messaging and video calling applications (e.g., Messenger, WhatsApp, Skype, FaceTime, etc.), and also the use of physical activity applications to measure their level of physical activity (e.g., the number of steps, calories burned, number of km traveled, etc.).

##### Level of Physical Activity

The validated short version (7 questions) of the “International Physical Activity Questionnaire” (IPAQ) was used [39]. This questionnaire assesses the total physical activity and sedentary time over the previous 7 days. The questionnaire covers vigorous activity, moderate activity, walking, and sitting (sedentary time), whether during leisure activities, at work, in daily life, or during transportation. The questionnaire classifies the subject according to 3 levels of activity: inactive, moderate, and vigorous [39]. The score is expressed as METs (metabolic equivalent of tasks) per minute/week.

##### Physical Activity Preferences

In order to create a specific and adapted physical activity plan, the physical activity preferences of the participants were ascertained, using the following questions:

“How many days a week would you be willing to be physically active?”, “How long do you think a physical activity session should last?”, “Do you prefer to perform physical activity alone, with your partner, with a friend/family member, or in a group?”, “Do you prefer to perform physical activity outdoors, in a sports facility, or at home?”, “What type of physical activity do you prefer?”, “What are your facilitators of physical activity?”, and “What are your barriers to physical activity?”. All these questions were closed questions, with the possibility of giving another open answer.

To assess the respondents’ preferences for online physical activity, participants were also asked the following questions: “Would you be willing to follow an online physical activity program over the internet?”, “Would you be willing to follow an online physical activity program using pre-recorded videos?”, “Would you be willing to follow an online physical activity program live, with a coach?”, “For how many days per week would you be willing to participate in an online physical activity program?”, “How long do you think an online physical activity session should last?”, “What type of online physical activity program would you be willing to participate in?”, “What are your facilitators of online physical activity?”, and “What are your barriers to online physical activity?”. All these questions were closed questions, with the possibility of giving another open answer.

##### Subjective Age and Age of Becoming Young or Old

Participants were asked to report how old they felt in years. We calculated the discrepancy between their subjective age and chronological age (discrepancy age = subjective age − chronological age). A positive value indicates an older subjective age, and a negative value indicates a youthful subjective age. In fact, feeling older or younger is an expression of subjective age [40,41].

Moreover, in order to assess the participant’s views on which periods of life correspond to youth and old age, they were asked to be specific (in age): “At what age does a person stop being young?” (end of youth) and “At what age does a person become old?” (the beginning of old age) [41,42].

##### Health Literacy

Comprehensive health literacy was measured using the validated HLS-EU-Q 16 questionnaire [43]. Each question of the HLS-EU-Q16 was answered by selecting one of the available options: “very difficult”, “difficult”, “fairly easy”, or “very easy”. The original version of the HLS-EU-Q16 measures health literacy in three domains: healthcare (seven items), disease prevention (five items), and health promotion (four items). Each of the 16 questions was coded as follows: “very difficult/fairly difficult” =  0, “fairly easy/very easy”  =  1). The total score (0 to 16 points) was divided into three categories of health literacy: “likely inadequate” (0–8 points), “likely problematic” (9–12 points), and “probably sufficient” (13 points) [44]. 

##### Sense of Coherence

The validated SOC-13 scale was used to assess the sense of coherence [45]; this can be defined as a permanent and reliable feeling of confidence [34]. The scale consists of items grouped in the following subscales: comprehensibility (understanding life events—five items), manageability (feeling that you can cope—four items), and meaningfulness (life making sense—four items). The final score of the scale for each participant score included a reverse score of questions as 1, 2, 3, 7, and 10 (where score 7 = 1, 6 = 2, 5 = 3, 4 = 4, 3 = 5, 2 = 6, and 1 = 7). The scores ranged from 13 to 91 points, with higher total scores indicating a greater sense of coherence. 

#### 2.3.2. Study Instruments

For this study, a self-administered questionnaire was developed by our team (i.e., including experts from different fields: geriatricians, psychologists, and physical activity and public health specialists). All the questions that were developed were closed questions with the possibility to give another open answer. This survey also included a number of validated questionnaires (SF-12, IPAQ, HLS-EU-Q 16, and SOC-13), as described above. The survey was pre-tested with 10 community-dwelling older adults to ensure that the questions were well-understood and easy to answer. The survey was designed to investigate the health determinants of people aged 65 years and over, particularly for informal caregivers. Other variables related to caregivers were also collected.

### 2.4. Statistical Analysis

Data were expressed as the mean and standard deviation for continuous variables and as a number and percentage for categorical variables. The analysis of the normality of the variables included the Shapiro–Wilk test, a comparison of means and medians (i.e., if the values are closed, the variable follows the normal distribution), and the generation of a Q-plot (i.e., if the points on the plot closely follow a straight line, this suggests that your data approximate a normal distribution) to assess the distribution of the data. Analyses were performed on all data available for each question. *t*-test (or Wilcoxon’s test for non-parametric variables) and chi-square tests (or Fisher for non-parametric variables) were used to compare the variables between informal caregivers and their peers. Results were statistically significant at the 5% critical level (*p* < 0.05). All calculations were carried out using RStudio 4.2.2.

## 3. Results

### 3.1. Study Participants

A total of 111 volunteers completed the survey. The mean age was 70.0 ± 3.8 years and 71.2% of the respondents were women. The majority (91.8%) of respondents were Belgian. The socio-demographic characteristics of the study participants are shown in Table 1.

### 3.2. Informal Caregivers

Of the 111 respondents, 90 (81%) answered the question about informal caregivers. Among this number, 30 out of 90 (33.3%) reported that they regularly helped a person with a loss of autonomy. The baseline characteristics of the informal caregivers (IC) were comparable to their peers (P) in terms of age (IC: 70.2 ± 3.8 y vs. P: 69.6 ± 3.6 y; *p* = 0.29), sex (ratio of women:men; IC: 7 (23.3%):23 (76.6%) vs. P: 19 (31.7%):42 (67.7%); *p* = 0.45), and BMI (IC: 28.0 ± 4.9 vs. P: 26.3 ± 3.1 kg/m²; *p* = 0.05).

Details on the roles of the informal caregivers are given in Table 2. In summary, most of the time, the care-receiver was a spouse (33.3%) or a parent/grandparent (33.3%). In 40% of the cases, the informal caregiver lived with the care-receiver. Otherwise, the caregiver lived, on average, 16 km away from the care-receiver. The average age of the care-receiver was 75.9 ± 12.7 years. The help provided mainly concerns administrative management (66.7%), medication management (55.6%), and supervision (51.9%). In addition, the care-receiver had physical difficulties (46.4% of the cases), mental difficulties (14.3% of the cases), or both physical and mental difficulties (39.3% of the cases). Almost half of the respondents (48.3%) helped the person 7 days a week, for a total of 11.9 ± 9.5 h per week on average. The respondents had been helping the person for 8.9 ± 7.2 years on average. Their personal motivations to help the care-receiver were mainly love/affection/friendship (82.1%), but 39.3% declared that it was a duty, while 28.6% were carers to keep a promise. Then, in 42.7% of cases, the respondent was the only person who cared for the care-receiver. If the respondent was not the only one caring for the person, they shared the task with another family member (76.5%) and/or with a health professional (47.1%). Finally, the Zarit burden was 61.9 ± 15.2/88, which indicates a mean score just reaching the severe burden level.

### 3.3. Quality of Life

**Figure 1:**
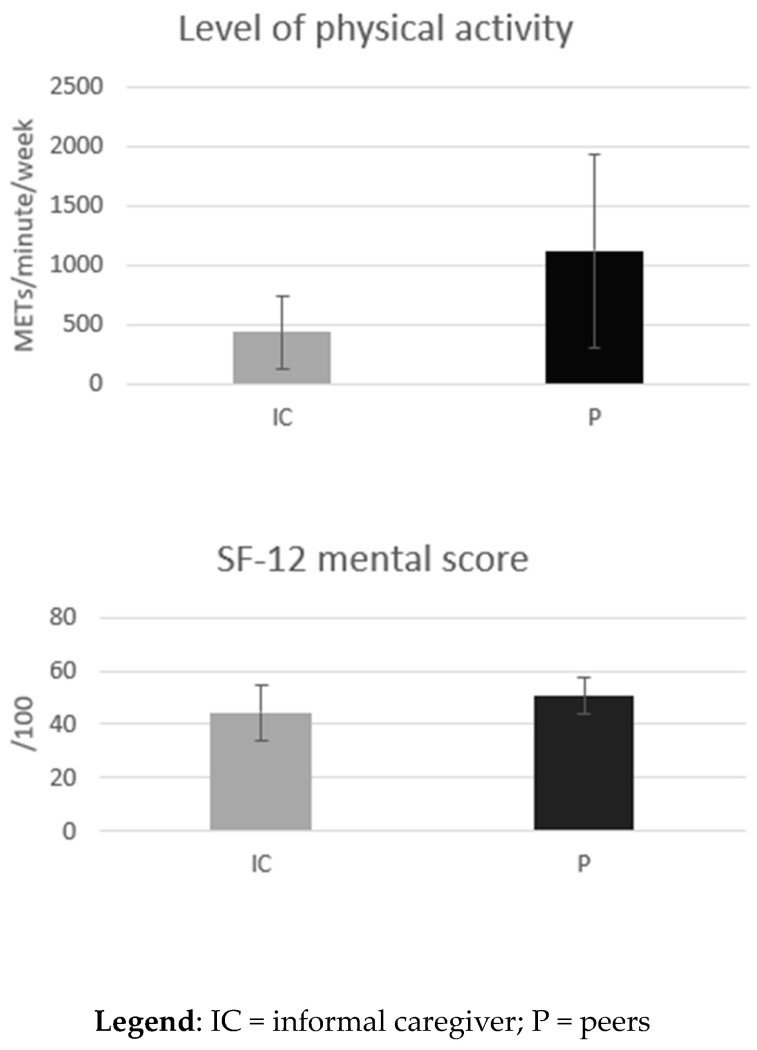
A summary of the significant differences in health determinants between informal caregivers and their peers.

Informal caregivers had a significantly lower mental score on the SF-12 than non-informal caregivers (IC: 44.3 ± 10.2 vs. P: 50.7 ± 7.0; *p* = 0.004). However, the physical score (SF-12) is comparable between the two groups (IC: 61.7 ± 31.9 vs. P: 47.4 ± 7.1; *p* = 0.11) (Figure 1).

### 3.4. Access to Technology

Access to technology was similar between informal caregivers and other older adults (*p*-values > 0.05 for all variables). Globally, more than 80% of informal caregivers use the internet every day and have a smartphone. Nevertheless, only a third of them have ever used an application for physical activity. According to the respondents, the most widely used applications for physical activity were YouTube and Strava (Table 3).

### 3.5. Level of Physical Activity

The level of physical activity, expressed in METS-minute per week, was significantly lower among the informal caregivers than among their peers (IC: ±289 (119–628) vs. P: ±819 (344–1523) *p* = 0.01) (Figure 1). This means that informal caregivers engaged in less physical activity. More specifically, 29.9% of the IC had engaged in vigorous physical activity in the last 7 days, compared to 49.2% of the P. The proportion of people who had performed moderate physical activity in the last 7 days was comparable in the 2 groups (IC: 72% vs. P: 70%). Among the IC, 81% had walked for at least 10 min in a row in the last 7 days, compared with 94.5% of the P. In addition, the proportion of subjects with a physical activity level below 600 METS-minute (i.e., inactive people) was higher among the informal caregivers than among their peers (IC: 12 (75%) vs. P: 15 (35.7%); *p* = 0.003).

### 3.6. Physical Activity Preferences

#### 3.6.1. Preferences for Face-to-Face Physical Activity

Most of the informal caregivers (98.8%) said that they were willing to engage in physical activity. More specifically, they were willing to perform physical activity for 2 (30.9%), 3–4 (38.1%) or >4 days (29.8%) per week. In addition, almost half of the informal caregivers believed that a physical activity session should last 60 min. It is also interesting to note that 63.4% of the informal caregivers preferred to perform physical activity in groups while 41.5% preferred to exercise alone. About a quarter of the respondents preferred to perform physical activity with their partner or with a friend/family member. In addition, 72% of the informal caregivers preferred to perform physical activity outdoors, 57.3% in sports facilities, and 25.6% at home. Among them, 61.3% preferred cardiovascular physical activities, 42.5% preferred mixed activities, 35% preferred body and mind activities, and 16.3% preferred strengthening activities. Informal caregivers reported that 53.1% of them would be willing to participate in a paid physical activity program, while 85.2% were willing to take part in a free program. In addition, 48.1% of them reported that they would be more motivated to perform physical activity if a coach were present. Barriers to and facilitators of physical activity are presented in Table 4, below.

#### 3.6.2. Preferences for Online Physical Activity

Among the informal caregivers, 31.7% were willing to follow an online remote physical activity program. Of these, 100% were willing to participate in an online remote physical activity program using pre-recorded videos, and 44.4% were willing to participate through a live physical activity program (with a coach). The majority of them were willing to participate in an online remote physical activity program 1 or 2 days a week. For almost 90% of these respondents, online remote physical activity was expected to last 30 min. In addition, 55.6% of the respondents were willing to take part in mixed activities, 33.3% in cardiovascular activities, 22.2% in body and mind activities, and 11.1% in strengthening activities. The main barriers to online remote physical activity identified in the present survey were (n = 9): lack of space at home (33.3%), fatigue (33.3%), fear of performing an exercise badly (22.2%), lack of social interaction (22.2%), lack of motivation (22.2%), incompatibility of schedules (11.1%), aging (11.1%), fear of injury (11.1%), and a lack of physical activities adapted to a particular age (11.1%). Facilitators of such a physical activity program were: the absence of travel (66.7%), the limited duration of the sessions (66.7%), the habit of using technology (55.6%), health benefits (55.6%), the possibility of being accompanied by a partner during the session (33.3%), and being in groups of people of the same age (11.1%).

### 3.7. Subjective Age and Age When Becoming Young or Old

In this survey, we found that both informal caregivers and others feel younger than their chronological age (IC: −7.2 ± 4.9 vs. P: −8.6 ± 7.4 years; *p* = 0.24). Both groups estimate that we become old at around 77 years (IC: 77.9 ± 7.5 vs. P: 77.5 ± 8.8 years; *p* = 0.45) and stop being young at around 65 years (IC: 65.5 ± 14.2 vs. P: 64.6 ± 16.6; *p* = 0.44).

### 3.8. Health Literacy

There was no difference between the two groups in terms of health literacy as assessed by the HLS-EQ-U16 (IC: 12.5 ± 3.2 vs. P: 12.7 ± 2.7; *p* = 0.23). However, the score in both groups was on the borderline between “likely problematic” and “sufficient”. More specifically, health literacy was “likely inadequate” in 19% of the IC and in 13.4% of the P, “likely problematic” in 23.8+ of the IC and in 26.9% of the P, and “sufficient” in 57% of the IC and in 59.6% of the P.

### 3.9. Sense of Coherence

The score for the sense of coherence was around 50 points out of 91 in both groups (IC: 50.7 ± 5.5 vs. P: 52.8 ± 9.4; *p* = 0.21), which represents an average score.

## 4. Discussion

The present survey aimed to better understand the health determinants of informal caregivers aged 65 years and over compared to those of their peers.

First, the results suggest that the mental component score for quality of life is lower among older informal caregivers than among their peers. This suggests that informal caregivers may experience more stress, worries, and emotional difficulties related to their caregiving role, which can have a negative impact on their overall well-being. This is consistent with a recent systematic review in which quality-of-life data indicated that a large proportion of informal caregivers experienced clinical levels of anxiety (33%) or depression (12–32%) [46]. These figures are even higher among the caregivers of people with mental disorders (e.g., dementia) [47]. Another qualitative systematic review also concluded that being an informal caregiver has a significant impact on quality of life [48]. It should be noted, however, that the studies included in these systematic reviews were not specific to older informal caregivers. Our study confirms the previous findings for a population of people aged 65 years and over. However, the results of a systematic review of longitudinal studies suggest a negative association between informal care and mental health in working-age adults [49]. Moreover, the informal caregivers reported a severe burden, which is associated with a risk of depressive symptoms and is also associated with care cessation and the admission to a nursing home of the care-receiver [50].

We then found that access to technology is similar between informal caregivers and other older adults. Informal caregivers have comparable accessibility to technological devices such as computers, smartphones, or tablets, compared to other older adults who are not informal caregivers. According to our results, 80% of informal caregivers use the internet every day and have a smartphone. This percentage is comparable to the findings reported by Shaffer et al. (77.5% of internet users among informal caregivers) [26]. This result is not surprising because the informal caregivers expressed a strong interest in technological innovations to support them in their caregiving role [26]. Despite this finding, only a third of our respondents had ever used an application for physical activity, although a recent study demonstrated the feasibility, usability, and acceptability of novel digital health apps for informal caregivers to improve their physical activity levels [51].

Moreover, physical activity is also recognized as an important determinant of health [52]. Our survey shows that most informal caregivers are inactive, and their levels of physical activity are much lower than those of their peers (with very significant differences). This suggests that the responsibilities and demands of caregiving may impede informal caregivers from engaging in regular physical activity. The substantial differences in physical activity levels highlight the potential negative impact of caregiving on the ability of informal caregivers to prioritize their own physical well-being. Caregiving duties may consume a significant amount of time and energy, leaving caregivers with limited opportunities to engage in physical activities or maintain an active lifestyle. The same finding was reported by Rokicka et al., who found that informal caregivers were generally more likely to allocate less time to physical activity, hobbies, and their own social life [53]. This can be explained by the fact that the role of an informal caregiver is time-consuming. Thus, informal caregivers reduce the time that they spend on leisure and social activities, including physical activity, leading to physical problems and reduced social well-being [54]. Therefore, it seems necessary to propose a physical activity intervention for informal caregivers to reduce the public health problem of physical inactivity behavior in this population. A systematic review of randomized controlled trials highlighted the fact that physical activity interventions significantly improved mental health but had inconsistent effects on physical health in the informal caregivers of older adults with chronic diseases [55]. The inconsistent results can be explained not only by the heterogeneity of the interventions proposed but also by the differences in adherence rates to physical activity programs.

Our survey will help us to offer physical activity interventions that best meet the expectations of informal caregivers, in an attempt to increase the adherence rate. Indeed, according to our survey, this physical activity should be delivered online using pre-recorded videos in order to address the main barriers to the practice of physical activity (i.e., lack of time, incompatibility of schedules, and distance). The program should take place 2 times a week for 30 min and should include mixed activities (i.e., cardiovascular, strengthening, and body and mind activities). This kind of physical activity program has been tested in previous studies and has shown encouraging results in older populations [56,57], but this needs to be confirmed in informal caregivers. The preferences of informal caregivers seem slightly different from those observed in other seniors (for example, a preference for pre-recorded videos compared to live videos in other seniors), which can be explained by the time they devote to the care-receiver.

Another determinant of health in older adults examined in our study is ageism. In our study, informal caregivers, like their peers, feel younger than their chronological age. The finding indicates that informal caregivers may possess a positive self-perception of their age. This finding is important because feeling younger than one’s chronological age is associated with positive outcomes, whereas feeling older than one’s chronological age is associated with negative outcomes [41,58,59]. Care should be taken when interpreting these data, as some authors find that the subjective age measure is a general concept and lacks information about the person’s actual experience of aging that underlies their perception [58]. Our results are in line with those observed in the general population. In a Danish sample, people older than 25 years had a younger subjective age [60]. More precisely, they felt 20% younger than their actual age (~10% in our population) [60]. The difference (20% vs. 10%) can be explained by the fact that our sample group was older. Recently, the subjective age was found to be 13.8 years lower than the chronological age among older adults (>65 years) in Norway [61]. This result is close to the one that we obtained. It is important to note that feeling less young was associated with poorer intrinsic abilities (i.e., vitality and autonomy) in the Norwegian study [61]. More relevantly, in a German study, it was highlighted that the beginning of informal care was significantly associated with a better attitude toward old age, which seems consistent with our results. However, in this study, the end of informal care was significantly associated with an increase in subjective age and an earlier onset of old age [29].

Health literacy enables people to access, understand, and evaluate health information. Health literacy is, therefore, an important determinant of health. In our population, health literacy was borderline between “likely problematic” and “sufficient” (among informal caregivers and non-informal caregivers). Our results confirm those from a Portuguese study showing that health literacy among informal caregivers was mostly sufficient [62]. According to the literature, female sex, older age, and lower education were independent predictors of low health literacy [32]. Thus, personalized support is needed for informal caregivers who are at a high risk of low health literacy [63].

The final health determinant examined in this study is the sense of coherence, which was comparable between informal caregivers and their peers. The finding suggests that despite the additional responsibilities and challenges associated with caregiving, informal caregivers have a similar capacity to understand and manage stressful situations as their non-caregiver counterparts. To the best of our knowledge, the sense of coherence of informal caregivers is poorly understood. However, it is an important determinant because a strong sense of coherence is associated with good perceived health and quality of life, whereas a weak sense of coherence is associated with caregiver burden and psychological distress, particularly depression and anxiety [64].

The results of this survey should be interpreted with caution since the sample is not representative of all the older informal caregivers (the study has a lack of statistical power because of the absence of sample size calculation). In addition, the survey was conducted on a voluntary basis and was mainly completed online. Therefore, there is a sampling bias. A recruitment bias is also acknowledged since we recruited participants during a special week dedicated to informal carers, so the study population may have been of healthier individuals if they could attend such events. Moreover, most of the respondents were Belgian. Furthermore, the link to the online survey was posted on the co-authors’ social media, which could have led to a recruitment bias. Results may vary considerably from one country to another since the role of informal caregivers varies according to culture and healthcare system (support). Since only people aged 65 and over were included in our research, further studies are needed to assess the health of informal carers of working age. In addition, not all health determinants were taken into account in this survey. For example, nutritional and diet status were not covered. A bias in the understanding of the questions is also possible, due to the online nature of the survey (absence of an investigator). Finally, this is a descriptive analysis and the relationship between health determinants has not been investigated, due to the low number of informal caregivers.

## 5. Conclusions

In conclusion, our study highlights that informal caregivers experience a lower quality of life compared to their peers. Furthermore, we observed that older informal caregivers have similar health determinants to their counterparts, except for lower levels of physical activity. Given the potential for public health interventions to address this issue, we recommend implementing a remote mixed physical activity program for older informal caregivers. This program, consisting of 30-min sessions twice a week, should be accessed online and tailored to meet the specific needs and constraints of this caregiving population.

The study’s implications for policy and intervention include the need for healthcare policies to recognize and support the well-being of informal caregivers. Adequate resources should be allocated to develop and implement targeted interventions that promote physical activity among older caregivers. Additionally, public health initiatives should prioritize increasing awareness about the importance of caregiver well-being and the availability of support programs. By addressing the barriers to physical activity and providing accessible resources, policymakers and healthcare professionals can improve the overall health and quality of life of informal caregivers, ultimately benefiting both the caregivers and the person they help.

## Figures and Tables

**Table 1:** Socio-demographic characteristics of the participants (n = 111).

Variables	n	Mean ± SD OR n (%)
Age (years)	111	70.0 ± 3.8
Sex (ratio men:women)	111	32 (28.8):79 (71.2)
BMI (kg/m^2^)	106	26.6 ± 3.6
**Country:**	109	
Belgium		100 (91.7)
Canada		9 (8.3)
**Marital status:**	110	
Married		55 (50)
Married since (years)	55	36.3 ± 13.5
Bachelor		9 (8.2)
Widower		26 (23.6)
Widower since (years)	26	13 ± 10.7
Divorced/separated		20 (18.2)
Divorced since (years)	20	19.4 ± 10.1
**Living place:**	98	
House		83 (84.7)
Apartment		13 (13.3)
Nursing home		2 (2)
**Number of people living with the respondent:**	98	
0		27 (27.6)
1		53 (54.1)
2		16 (16.3)
3		2 (2)
**Highest level of education:**	97	
University		19 (19.6)
Higher education		30 (30.9)
Upper secondary education		31 (32)
Lower secondary education		14 (14.4)
Primary education		1 (1)
None		2 (2.1)
**Monthly household income:**	95	
>EUR 3000		29 (30.5)
EUR 2000–3000		19 (20)
EUR 1000–2000		32 (33.7)
<EUR 1000		0 (0)
Refusal to answer		13 (13.7)
Do not know		2 (2.1)
Smoker (yes)	97	4 (4.1)
Number of cigarettes per day	4	18.3 ± 7.7
**Number of alcoholic drinks:**	93	
0 per week		25 (26.9)
1 or 2 per week		35 (37.6)
1 or 2 per day		26 (28)
>2 per day		7 (7.5)
Number of prevalent chronic diseases	93	1.8 ± 1.4
Number of drugs consumed per day	93	3.1 ± 2.2
Number of vitamins or food supplements consumed per day	91	1.6 ± 1.2

**Table 2:** Details regarding the role of informal caregivers (n = 30).

Variables	n	Mean ± SD OR n (%)
**The care-receiver is:**	30	
The spouse		10 (33.3)
A parent/grandparent		10 (33.3)
A child		2 (6.7)
A friend		5 (16.7)
A neighbor		2 (6.7)
Other		1 (3.3)
The care-receiver and the caregiver live together (yes)	30	12 (40)
Distance between the homes of the care-receiver and the caregiver (km)	18	16.3 ± 21.1
Age of the care-receiver (years)	27	75.9 ± 12.7
**Tasks with which the care-receiver is helped:**	27	
Basic activities of daily living (e.g., toileting, dressing, and mobility)		6 (22.2)
Meals		12 (44.4)
Household		13 (48.1)
Laundry		10 (37.0)
Budget management		17 (63)
Administrative management		18 (66.7)
Drug management (e.g., preparation of the drugs)		15 (55.6)
Work		1 (3.7)
Hobbies		13 (48.1)
Social relations		11 (40.7)
Transport		20 (74)
Surveillance		14 (51.9)
**What is the care-receiver suffering from?**	28	
Physical difficulties		13 (46.4)
Mental difficulties		4 (14.3)
Physical and mental difficulties		11 (39.3)
**Number of days per week devoted to the care-receiver:**	29	
<1 day/week		5 (17.2)
1–2 days/week		5 (17.2)
3–4 days/week		5 (17.2)
5–6 days/week		0 (0)
7 days/week		14 (48.3)
Number of hours per week devoted to the care-receiver (hours)	27	11.9 ± 9.5
How long the caregiver has been caring for the person (in years)	29	8.9 ± 7.2
**Personal motivations for caregiving:**	28	
Love, affection, or friendship		23 (82.1)
Acknowledgment		2 (7.1)
The challenge		0 (0)
Obligation		1 (3.6)
Keeping a promise		8 (28.6)
Duty		11 (39.3)
Religious beliefs		1 (3.6)
Financial constraints		0 (0)
Other		1 (3.6)
Is the carer the only one caring for the person? (yes)	29	12 (42.7)
**Another person who helps him/her:**	17	
Family member(s)		13 (76.5)
Friend(s)		0 (0)
Health professional(s)		8 (47.1)
Zarit burden (/88)	24	61.9 ± 15.2

Note that the number of informal caregivers changes from one variable to another, depending on the total volume of data available. The participants did not always answer all questions.

**Table 3:** The respondents’ access to technology.

	Informal Caregivers (n = 30)		Peers (n = 60)		
Variables	n	Mean ± SD OR n (%)	N	Mean ± SD OR n (%)	*p*-Value
**Use of the internet:**	27		58		0.23 ^1^
Never		2 (7.4)		1 (1.7)	
Every month		1 (3.7)		2 (3.5)	
Every week		2 (7.4)		4 (6.9)	
Every day		22 (81.5)		51 (87.9)	
Use of the internet for how many years	24	19.6 ± 5.9	54	18.8 ± 6.5	0.34 ^2^
**Do you have the following connected objects?**	26		57		0.19 ^1^
A smartphone		22 (84.6)		54 (94.7)	
A tablet		17 (65.4)		29 (50.9)	
A laptop		16 (61.5)		40 (70.2)	
A computer		12 (46.2)		22 (38.6)	
How long have you owned these connected objects?	24	17.5 ± 7.4	55	17.1 ± 6.8	0.45 ^2^
Do you regularly use messaging and video-calling applications (e.g., Messenger, WhatsApp, Skype, FaceTime, etc.)? (yes)	26	21 (80.7)	58	52 (89.7)	0.30 ^1^
Have you ever used an application(s) for physical activity? (yes)	26	8 (30.8)	58	22 (37.7)	0.63 ^1^
Have you ever used an application(s) to measure your level of physical activity (number of steps, calories burned, number of kilometers traveled, etc.)? (yes)	26	15 (57.7)	57	25 (43.9)	0.41 ^1^

Statistical tests used: ^1^ Fisher’s test; ^2^ *t*-test.

**Table 4:** Facilitators of and barriers to physical activity according to the informal caregivers (n = 25).

Barriers to Physical Activity: n (%)		Facilitators of Physical Activity: n (%)	
Cost of physical activities	7 (28)	Health benefits	20 (80)
Lack of time	9 (36)	Previous sports participation	2 (8)
Schedule constraints	6 (24)	Free of charge	13 (52)
Aging (e.g., “I am too old to exercise”)	2 (8)	Proximity to home	11 (44)
Health condition	3 (12)	Limited duration of the sessions	9 (36)
Fatigue	10 (40)	Atmosphere (e.g., conviviality of the group)	16 (64)
Distance (e.g., far from home, access to public transport, or inability to drive)	4 (16)	Groups of people of the same age	10 (40)
Fear of getting involved	5 (20)	Supervision of the sessions by a professional	11 (44)
Fear of getting injured	0 (0)	Opportunities for physical activity at home	6 (24)
Lack of physical activities adapted to a particular health condition	2 (8)	The possibility of being accompanied by a partner during the session	3 (12)
Lack of physical activities adapted to a particular age	2 (8)	Other	0 (0)
Lack of motivation	8 (32)		
Impossibility of leaving the person that is being helped on their own	2 (8)		
Other	1 (4)		

## Data Availability

Data available on request.
